# Risk factors for unexpected death in patients identified by a communication and resolution program

**DOI:** 10.3389/frhs.2025.1712574

**Published:** 2025-11-21

**Authors:** Peter Lodato, Neal D. Goldstein, Alexandra M. Mapp, Adebayo Gbadebo, Stephen A. Pearlman

**Affiliations:** 1 Department of Patient Safety and Accreditation, ChristianaCare, Newark, DE, United States; 2 Department of Epidemiology and Biostatistics, Dornsife School of Public Health, Drexel University, Philadelphia, PA, United States; 3 Department of Microbiology and Immunology, College of Medicine, Drexel University, Philadelphia, PA, United States; 4 Institute for Research on Equity and Community Health (iREACH), ChristianaCare, Newark, DE, United States; 5 Department of Clinical Effectiveness, ChristianaCare, Newark, DE, United States; 6 Department of Pediatrics, Sidney Kimmel Medical College, Thomas Jefferson University, Philadelphia, PA, United States

**Keywords:** CANDOR, CRP, communication and resolution, disparities, patient safety, SDOH, patient harm, social drivers of health

## Abstract

Many patients experience unexpected harm while receiving healthcare, with a lasting impact on patients, families, and caregivers. Communication and Resolution Programs are being adopted with increased frequency, as a more systematic, transparent, and equitable approach to these unexpected outcomes. The aim of this study was to identify whether demographic factors played a role in identifying patients with unexpected death, as managed in our CRP. This nested case-controlled compared 236 patients who experienced an unanticipated death with 2,360 controls who died expectedly over a 10-year period. Patients with unexpected death were more likely to be Black (AOR 2.18 95% CI 1.01–4.68), higher comorbidity burden (AOR 1.07 per additional co-morbidity, 95% OR 1.01–1.14), and a lower Relative Expected Mortality (AOR: 5.39; 95% CI: 1.76–16.55). Awareness of these demographic risk factors for unexpected mortality may lead to changes in how these patients are evaluated and treated. Communication and Resolution Programs can be used to identify the patients at the highest risk for unexpected outcomes.

## 1 Introduction

Despite a call to arms over 25 years ago in the report “To Err is Human,” medical errors resulting in patient harm continue to vex our healthcare system (1). In the ensuing decades, robust efforts have been undertaken to make care processes safer, yet the US continues to annually see as many as 250,000 deaths and an additional 400,000 hospitalized patients experiencing preventable harm (2–4). According to some studies, the volume of medical errors would rank it as the third leading cause of death in the US, only behind heart disease and cancer (5, 6). These adverse events result in increased healthcare costs estimated between 20 and 45 billion dollars annually, and frequently have lasting detrimental effects on patients, families, and the caregivers involved (2, 3, 7, 8). This undesirable distinction has rightly elevated medical errors and patient harm as a serious public health problem, warranting further study into the ways in which it can manifest and the factors that may influence the risk of harm.

Concurrently, much attention in the past decade has been given to the social drivers of health (9–16). A recent systematic review by Chauhan and colleagues, showed that patients from ethnic minorities were at higher risk of patient safety events (17). There are many contributing factors to explain this phenomenon, including low socioeconomic status, higher burden of chronic disease, limited health literacy, and feelings of disempowerment (18–20). Limited English proficiency may impact a patient's ability to raise important concerns about their care (21, 22). Taken collectively, these factors may impact both the quality of care and the healthcare outcomes for these marginalized groups of patients. While there is evidence that marginalized groups have a higher risk of being involved in patient safety events, it is less well understood how effectively harm events involving these populations are identified and shared with the involved patients and their families.

Given the high volume of harm events that has been established, it is imperative that health systems have effective process for responding to these occurrences. Communication and Resolutions Programs (CRP) are a more transparent and equitable approach, in which health systems proactively seek to identify, disclose, analyze, and share the details of harm events with patients and their families (23, 24). High-reliability organizations with fully implemented CRPs typically foster a culture in which unexpected outcomes are regularly reported to quality, safety, and/or risk management teams, so that opportunities for the implementation of risk reduction strategies and quality improvement can be pursued. Additionally, for cases in which the analysis determines the standards of care were not met, and that the care contributed to the unexpected outcome, a mature CRP will proactively offer fair compensation to those affected.

While their origins date back more than two decades, more recently they are becoming the standard of care (24, 25). Among the earliest reports of CRPs, the Veteran's Administration Hospital in Lexington, Kentucky reported on a method of earlier resolution involving two cases with severe adverse outcomes, thereby obviating the need for relying on a slow and costly legal system (26). Soon thereafter, the University of Michigan published their results using the CRP approach, demonstrating a significant impact on the medicolegal outcomes, including significant financial benefits owing to smaller settlements and faster resolution (24, 27, 28).

The impact of the CRP approach goes beyond the benefits in the malpractice realm. In the previous “deny and defend” approach, clinicians and caregivers were discouraged from discussing any aspects of the case with anyone, including their closest confidants (27, 29, 30). This led to what is often characterized as the “second victim” phenomenon (31–33). It would not be uncommon for healthcare professionals involved in adverse events to suffer in silence, resulting in a range of negative mental health impacts such as anxiety, depression, post-traumatic stress disorder, and some going so far as to leave the healthcare profession and even self-harm (34–43). Using the more enlightened approach of CRP allows for more open, inclusive discussion and provides the opportunity to offer support to the medical team. An additional benefit to the CRP process is a core focus on system learning from the case review. By looking at serious safety events through the lens of process improvement, healthcare organizations can identify opportunities for the delivery of safer medical care and implement mitigating strategies to significantly decrease the risk of future safety issues.

Recognizing the benefits of the CRP approach, in 2013 President Obama appropriated $23 million in funding to the Agency for Healthcare Research and Quality (AHRQ) to perform a demonstration project (44–47). Three hospital systems, including ChristianaCare (Newark, Delaware), participated in this pilot to develop a toolkit for other institutions to develop their own CRP. The resulting toolkit was subsequently made available on the AHRQ website (48). At our organization, our inclusion criteria for events requiring a system level CRP review are those in which an unexpected death or permanent harm occurred (defined below). Since beginning our program in 2015, we have reviewed over 650 events, of which 2/3 involved an unexpected mortality.

Given the evidence suggesting that individuals from marginalized populations are at a higher risk for unanticipated medical outcomes, and that an established CRP program ought to identify cases in which patients have experienced such outcomes, we sought to evaluate whether sociodemographic characteristics predicted unexpected death for patients managed through our CRP.

## 2 Materials and methods

### 2.1 Study design and setting

We conducted a nested case-control study to examine factors associated with the risk of unexpected death within ChristianaCare, a multi-hospital system located in the Mid-Atlantic region. ChristianaCare's Institutional Review Board reviewed and approved this study.

### 2.2 Study sample

Cases which met the criteria of unexpected mortality were selected from our CRP event registry from July 2015 to December 2023. Patients were excluded if they were under 18 years of age or admitted to Women's and Children's or Obstetrics services based on a low incidence and different criteria for these populations.

Eligible control patients were derived from a pool of general population non-CRP patients seen at the healthcare system and who expired during the same timeframe. We excluded control patients based on the same exclusion criteria utilized for our cases. Each CRP case was matched with up to 10 control patients using risk-set sampling on discharge month (±1 month, a proxy metric for death date), and length of stay (LOS) category. LOS categories were based on quartiles with the following categories: LOS < =1 day, LOS 2–4 days, LOS 5–7 days, LOS > =12 days.

### 2.3 Data collection

Data for this study were extracted through our hospital administrative data warehouse and from Vizient Inc., a third-party comparative, national, clinical database that utilizes discharge and line-item data for performance metrics and benchmarking (49). In addition, Vizient data provides risk-adjusted metrics for length of stay, morbidity, and cost (49).

#### 2.3.1 Outcome

The primary outcome of interest was unexpected patient death, as determined through our CRP review process. Unexpected mortality was established by medical event review teams that reviewed all mortalities and determined whether the death was expected or not based on clinical factors.

#### 2.3.2 Independent variables

We focused on the following sociodemographic characteristics as our primary exposures of interest: age (years), gender (male or female), race (White, Black/African American, and Other), ethnicity (Hispanic or Non-Hispanic), payer (private, Medicare, Medicaid, self-pay, and other), and Area Deprivation Index (ADI), an area-level ranking a neighborhood's socio-economic disadvantage. Patient addresses were geo-coded and linked to their census block ADI ranking. For this study, we utilized a state-level ADI index that ranged from 1–10, with 1 being the lowest area of need and 10 being the highest (50). ADI was categorized into “low” (ADI < =4), “low-medium” (ADI 5–6), “medium” (ADI 7–8), and “high” (ADI 9–10).

#### 2.3.3 Covariates

To account for confounding, we included several variables that related to patient acuity and characteristics of the hospitalization. Clinical covariates included admission to an Intensive Care Unit (ICU) and ICU length of stay (days). Comorbidities were assessed using the Elixhauser Comorbidity Index (51). This index is a measure of thirty comorbidities, weighted equally, which can be used to predict mortality and utilization of healthcare resources (51, 52). Proxy measures for patient risk were assessed using Vizient's risk adjusted or expected values, based on clinical characteristics, for mortality (49). Vizient's Relative Expected Mortality (REM) measures patient severity through risk mortality relative to their model cohort (49). Rankings are assessed as “well-below”, “below”, “slightly below”, “similar”, “slightly above”, “above”, or “well-above” expected mortality relative to other patients in their model cohort (49).

### 2.4 Statistical analysis

Descriptive statistics were used to summarize baseline characteristics of the CRP and control groups. Continuous variables, reported as mean ± standard deviation (SD), were compared using unpaired student t-tests, while categorical variables, reported as frequencies and percentages, were analyzed using Pearson's *χ*2 test.

To evaluate associations between patient characteristics and unexpected death in the matched sample, we used conditional logistic regression. Conditional logistic regression was performed using PROC LOGISTIC in SAS (version 9.4, Cary, NC), with a STRATA statement included to account for matched sets. Covariates that met a threshold *p*-value of ≤0.05 in descriptive analysis were included in the final model (age, race, payer, ICU admission, Elixhauser Comorbidity Count, ADI category, and Vizient's REM). Continuous variables (age and Elixhauser Comorbidity Count) were modeled per one-unit increase. Results were reported as adjusted odds ratios (AORs) with corresponding 95% confidence intervals (CI). We include E-values for our main findings. The E-value represents the minimum amount of uncontrolled confounding necessary to wholly explain the observed association (53). A two-tailed *p*-value of <0.05 was considered statistically significant.

## 3 Results

### 3.1 Participants

A total of 236 CRP patients and 2,360 patients in the control group were included in the analysis. Baseline characteristics (demographic and clinical) for the study population are presented in Table 1. CRP patients were more likely to be Black/African American (39% vs. 22.9%) and significantly younger than the control group, 63.1 years, (13.1) vs. 69.7 years, (15.8), respectively (*p* < 0.001 for both). CRP patients were less likely to have Medicare (47.9%) than the control group (68.0%) (*p* < 0.001). CRP patients had a higher mean comorbidity count than control patients, 8.2 (5.2) vs. 7.1 (5.0), respectively (*p* = 0.002).

**Table 1:** Demographic and clinical characteristics (CANDOR v Non-CANDOR) 2015–2023.

Characteristics	Cases	Controls	*p*-value
	(*N* = 236)	(*N* = 2,360)	
Age, yrs., mean (SD)	63.1 (13.1)	69.7 (15.8)	<0.001*
Race, *n* (%)			<0.001*
White	137 (58.3)	1,634 (73.0)	
Black/AA	92 (39.2)	513 (22.9)	
Other	6 (2.6)	93 (4.2)	
Ethnicity, *n* (%)			0.25
Hispanic/Latino	5 (2.2)	79 (3.6)	
Not Hispanic/Latino	226 (97.8)	2,099 (96.4)	
Payer, *n* (%)			<0.001*
Medicare	113 (47.9)	1,590 (68.0)	
Medicaid	20 (8.5)	216 (9.2)	
Private	39 (16.5)	226 (9.7)	
Self-pay	6 (2.5)	73 (3.1)	
Other	58 (24.6)	232 (9.9)	
Gender, *n* (%)			0.57
Male	124 (52.5)	1,286 (54.5)	
Female	112 (47.5)	1,074 (45.5)	
Mean Elixhauser Comorbidity Count, (SD)	8.2 (5.2)	7.1 (5.0)	0.00*
ICU Stay, *n* (%)			<0.001*
Yes	158 (67.0)	1,837 (77.8)	
No	78 (33.1)	523 (22.2)	
Mean ICU LOS, in days, (SD)	4.6 (5.8)	4.2 (6.3)	0.40
ADI, *n* (%)^a^			<0.001*
Low (ADI < =4)	36 (35.3)	861 (50.4)	
Low-Medium (ADI 5–6)	11 (10.8)	53 (3.1)	
Medium (ADI 7–8)	25 (24.5)	370 (21.7)	
High (ADI 9–10)	30 (29.4)	424 (24.8)	
Length of Stay—Categorical, *n* (%)			>0.99
0–1 days	66 (28.0)	660 (28.0)	
2–4 days	50 (21.1)	500 (21.1)	
5–11 days	65 (27.6)	650 (27.6)	
12+ days	55 (23.3)	550 (23.3)	
Relative Expected Mortality, *n* (%)			<0.001*
Well Below	8 (6.4)	67 (2.9)	
Below	26 (20.6)	228 (9.7)	
Slightly Below	12 (9.5)	72 (3.1)	
Similar	18 (14.3)	219 (9.4)	
Slightly Above	11 (8.7)	197 (8.4)	
Above	18 (14.3)	611 (26.1)	
Well Above	33 (26.2)	946 (40.4)	

a ADI is interpreted from lowest area of deprivation (1) to highest (10).

* Denotes statistical significance *p* < 0.05.

Clinically, CRP patients were less likely to be admitted to the ICU (67.0%) than patients in the control group (77.8%) (*p* < 0.001). REM also differed significantly between the CRP and control groups. CRP patients were less likely to be “well-above” their REM (26.2%) than patients in the control group (40.4%) (*p* < 0.001).

Our CRP program tracks metrics related to transparency (family meetings), as well as system improvement recommendations resulting from case identification and analyses. Of the 236 CRP patients included in this study, meetings were offered proactively to all patient families with whom our team had successfully established contact. Of those, meetings occurred with 115 families, in which the results of the CRP review were explained and families were offered an opportunity to ask questions and share their feedback.

Additionally, of the 236 CRP cases, 83 were identified to have at least one, if not more potential risk reduction/system improvement strategies. Approximately 184 strategies were proposed, evaluated, and/or implemented following CRP analyses. Expanding beyond the 236 cases used for this study, the analyses from our approximately 677 cases identified 243 cases with opportunities for risk reduction/system improvement strategies, for a total of approximately 567 possible improvements.

### 3.2 Multivariate logistic regression

Odds of unexpected death were 2.18 times higher in Black patients, when compared to White patients (AOR: 2.18; 95% CI: 1.01–4.68). Although not statistically significant, patients categorized as “Other” had 2.37 times higher odds of unexpected death compared to White patients (AOR: 2.37; 95% CI: 0.50–11.17).

Higher comorbidity burden was significantly associated with unexpected death. Specifically, for each one-unit increase in Elixhauser Comorbidity Count there was a 1.07 increase in the odds of unexpected death (95% CI: 1.01–1.14).

A REM classified as “below” the model cohort was significantly associated with higher odds of unexpected death compared to a “similar” REM classification (AOR: 5.39; 95% CI: 1.76–16.55). Conversely, patients with a REM classified “above” their model cohort had lower odds of unexpected death compared to those with a “similar” REM (AOR: 0.27; 95% CI: 0.08–0.99).

A complete summary of model results, including adjusted odds ratios (AOR) and 95% confidence intervals, is presented in Table 2.

**Table 2:** Adjusted odds ratios from conditional logistic regression (*N* = 2,596).

Covariate	HR	95% CI
Age (per 1 year increase)	0.98	0.95–1.01
Race		
Black vs. White	2.18	1.01–4.68
Other vs. White	2.37	0.50–11.17
Payer		
Medicaid vs. Medicare	0.91	0.19–4.50
Other vs. Medicare	2.18	0.90–5.31
Private vs. Medicare	2.62	0.98–7.02
Self-pay vs. Medicare	0.40	0.02–7.80
ICU Stay		
Yes vs. No	0.76	0.40–1.45
Elixhauser Comorbidity Count (per 1 unit increase)	1.07	1.01–1.14
MS-DRG REM Category		
Well Below vs. Similar	1.70	0.39–7.48
Below vs. Similar	5.39	1.76–16.55
Slightly Below vs. Similar	2.28	0.63–8.31
Slightly Above vs. Similar	0.71	0.19–2.71
Above vs. Similar	0.27	0.08–0.99
Well Above vs. Similar	0.55	0.19–1.59
ADI Category		
Low vs. Low-Medium	0.23	0.07–0.78
Medium vs. Low-Medium	0.22	0.06–0.83
High vs. Low-Medium	0.11	0.03–0.45

### 3.3 Sensitivity analysis

The E-value for the point estimate of Black race was 3.78 and for the lower bound of the confidence interval was 1.11. Similarly, for the Elixhauser and REM scores, the respective E-values for the point estimates were 1.34 and 10.25 and for the confidence interval were 1.11 and 2.92.

## 4 Discussion

Decades of research and quality improvement efforts have not succeeded in reducing patient harm (1). There have been pockets of progress, for example, the reduction of central line associated blood stream infections in adult patients (54–56). Yet other complications of care continue to trouble clinicians and the healthcare system in general. These unfortunate outcomes may occur for a variety of reasons including breakdowns in communication between various members of the healthcare team and diagnostic errors (57–68). Social determinants of health are among those factors identified by researchers as having an association with patient harm events.

What has begun to change is the response to events involving patient harm. Although Communication and Resolution Programs have existed for over two decades, the recent CMS Patient Safety Structural Measure has codified the need for hospitals to implement CRP as the standard approach to unexpected patient harm (24–26, 69, 70). Over the past 10 years, our organization has implemented a CRP approach for cases involving permanent harm or unexpected death, thus providing us with a robust population for additional analysis. For the purposes of this study, a subset of patients who experienced unexpected death was selected with a matched group who also experienced death while in the hospital that was deemed expected. Logistically, it would be more challenging to match controls to the group of patients with permanent harm.

Our results show that race predicted a patient being identified through a Communication and Resolution Program with an unexpected death. In our CRP population, Black patients were more than twice as likely to experience unexpected death compared to the control population. This was the case in both a univariate analysis and logistic regression, although uncontrolled confounding could also explain this result. Our cases were identified by discipline-specific event review teams with additional expertise in patient safety. Interestingly, Thomas and colleagues found that White patients had harmful safety events reported by staff more often than Black patients (71). They also reported racial differences in the types of events reported. The contrast with our study results may be attributable to the way by which our cases were identified. Our results are supported by a study by Ly and colleagues which found a higher incidence of patient safety events in Black Serving Hospitals compared to non-Black Serving Hospitals (72). A review of 24 papers on the racial and ethnic disparities in patient safety events by Okoroh et al, showed mixed results (73). The variation in geography and hospital level quality of care may account for the inconclusive nature of this literature review. Our study is the first to demonstrate racial differences in patients identified through a Communication and Resolution Program.

There are several reasons why race may play a role in patients identified as experiencing an unexpected death. Chauhan's systematic review of 45 articles found that marginalized populations have a higher risk of hospital acquired infections, adverse drug events, and overall complications (17). These differences may be driven by language difficulties, beliefs about illness and treatment, and patient engagement. Beliefs can impact compliance, as patients who mistrust the healthcare system are less likely to follow care plans. Patients from marginalized populations may have decreased family support. Other factors identified by Chauhan include lower socioeconomic status (SES) and insurance status. Our study did not show an impact of Area Deprivation Index, nor did we note any increased risk in Medicaid patients. Chauhan's review mentions an increased burden of illness, lower health literacy, a sense of disempowerment, and decreased access to care as additional vulnerabilities of marginalized populations. We noted an increased co-morbidity count in our patients with unexpected death but did not study the other factors. Coffey et al. noted that Black patients were more likely to develop complications, such as hospital acquired infections, post-operative sepsis, pressure injuries, post-operative respiratory failure, pulmonary embolism, and deep vein thrombosis (74). Many of these patient safety indicators could contribute to the unexpected death of a patient, hence increasing the risk for Black patients.

The Elixhauser Co-Morbidity Count was the second metric that predicted unexpected death in the multivariate model. Patients who experience unexpected death, had a higher burden of co-morbid conditions. This may seem counterintuitive initially but may reflect that these patients were sicker than initially recognized. This occurred even though the determination of expectedness of the death was made by clinicians experienced in event review. Perhaps, the fact that these patients were younger, was a greater determining factor in classifying the death as unexpected despite their higher co-morbidity count. Another possible explanation for this phenomenon is that patients with more co-morbid conditions have a higher likelihood of an unexpected deterioration and/or uncontrolled confounding.

The Relative Expected Mortality was the third factor that predicted unexpected death in the multivariate model. This correlation indicates that patients with a low REM were more likely to have their death classified as unexpected. This result supports the evaluation process performed by the teams which deemed the deaths to be unexpected. We note this was the strongest observed association and would require substantial uncontrolled confounding to negate.

Several factors correlated with unexpected death in the univariate analysis only but are still worth noting. Patients below age 65 or lacked Medicare were more likely to experience unexpected death, perhaps due to the perception that patients who are younger are often healthier. Fewer patients with unexpected mortality had stays in the ICU. This may be due to a perception that ICU patients carry a higher expectation of death than patients cared for in a non-ICU setting.

Our study has both strengths and limitations. One of the strengths is our rigorous statistical analysis including the use of incidence density sampling with 10 matched controls per case. The limitations include the use of administrative data which may have inaccuracies or not include relevant characteristics. In addition, linking patient data utilizing unique identifiers such as the medical record number (MRN) did not yield a one-to-one match between the initial case list and the final analytic dataset. This discrepancy was not due to study-defined exclusion criteria but rather to incomplete or inconsistent identifiers that prevented linkage and inclusion in the final analysis. We acknowledge the subjective nature of the determination of expected vs. unexpected deaths, even though these designations were made by teams with diagnostic and patient safety expertise.

The information gathered in our study can help clinicians, caregivers, and hospitals to better understand the risk of unexpected mortality. It may assist in the assessment of certain patient populations, increase vigilance for unexpected outcomes, and help to remove barriers that may account for the differences we noted. Suurmond and colleagues' qualitative study in the Netherlands noted that presumptions about cultural background resulted in patient safety events (75). Greater awareness about cultural differences may reduce some of the disparities identified in our study. Additionally, these data may assist clinicians with how to discuss these events with families. Olazo et al. noted that clinicians and caregivers have more difficulty disclosing patient safety events when they involve patients from marginalized populations (76).

Our study also highlights the additional transparency and quality improvement benefits that arise from fully implemented CRP programs. Through the identification, disclosure, and analyses of these critical events, organizations can create mechanisms to close quality gaps in the health system and share with families what has been learned about their loved one's care. In our study population, we identified 184 potential opportunities to improve the delivery of care in our health system, though this figure is likely an underestimate that does not account for strategies identified during separate but parallel review processes like Peer Review or Morbidity and Mortality conferences. Beyond the population included in this study, our CRP process has led to the identification of 567 potential risk reduction strategies related to the full 675 cases managed by the program.

Unfortunately, one of the key factors often identified in the analysis of adverse health outcomes is poor communication. This can be painfully true for marginalized populations. A lack of transparency following a serious adverse outcome, such as an unexpected patient death, can compound that injury and amplify the grief a family is experiencing. Conversely, fully implemented CRP programs create mechanisms for organizations to identify and acknowledge these adverse events, and to create bi-directional, transparent communication with the impacted next of kin. Family meetings are offered proactively, as a standard part of our process, to all families we can connect with, rather than waiting for families to request a meeting. In our study population, we coordinated meetings with 115 of the 236 patient families, during which we explained what we had learned through our analytic process, shared details of any quality improvement work that had resulted from the event, and in those instances where our analyses determined that the care contributed to the outcome, offer families financial support and compensation (separate analyses determined that the care may have contributed to the outcome of roughly 15% of our overall CRP caseload). Some factors identified in those cases where family meetings did not occur include: families no longer responding to outreach attempts, family members not having ongoing questions or concerns, not feeling there was value to meeting, expressed plans to pursue legal action, and still experiencing grief and not wishing to revisit the event.

There are other aspects of CRPs that are worthy of further study. In particular, identifying specific contributory factors which led to an unexpected outcome and whether these vary in different demographic groups would be of interest. This would allow for stratified comparisons between groups with different racial or socioeconomic characteristics who may be more at risk for communication lapses, diagnostic errors, treatment delays, or other mechanisms. This is a fertile area for additional research.

## 5 Conclusion

Patients who (1) identify as Black (2) have a higher burden of disease and (3) lower relative expected mortality have a higher rate of unexpected mortality. Recognition of these risk factors may result in differences in the assessment and treatment of these patients. Communication and Resolution Programs not only improve communication with patients and families about safety events but can also be used to identify the patients at the highest risk for unexpected outcomes.

## Data Availability

The data analyzed in this study is subject to the following licenses/restrictions: The data presented in this article is not readily available given the nature of this research. Requests to access the data should be directed to Dr. Stephen Pearlman at spearlman@christianacare.org.
